# Grounding Abstractness: Abstract Concepts and the Activation of the Mouth

**DOI:** 10.3389/fpsyg.2016.01498

**Published:** 2016-10-10

**Authors:** Anna M. Borghi, Edoardo Zarcone

**Affiliations:** ^1^Psychology, University of BolognaBologna, Italy; ^2^Institute of Cognitive Sciences and Technologies, Italian National Research CouncilRome, Italy

**Keywords:** abstract concepts, mouth activation, abstract words, language grounding, embodied cognition, grounded cognition

## Abstract

One key issue for theories of cognition is how abstract concepts, such as freedom, are represented. According to the WAT (Words As social Tools) proposal, abstract concepts activate both sensorimotor and linguistic/social information, and their acquisition modality involves the linguistic experience more than the acquisition of concrete concepts. We report an experiment in which participants were presented with abstract and concrete definitions followed by concrete and abstract target-words. When the definition and the word matched, participants were required to press a key, either with the hand or with the mouth. Response times and accuracy were recorded. As predicted, we found that abstract definitions and abstract words yielded slower responses and more errors compared to concrete definitions and concrete words. More crucially, there was an interaction between the target-words and the effector used to respond (hand, mouth). While responses with the mouth were overall slower, the advantage of the hand over the mouth responses was more marked with concrete than with abstract concepts. The results are in keeping with grounded and embodied theories of cognition and support the WAT proposal, according to which abstract concepts evoke linguistic-social information, hence activate the mouth. The mechanisms underlying the mouth activation with abstract concepts (re-enactment of acquisition experience, or re-explanation of the word meaning, possibly through inner talk) are discussed. To our knowledge this is the first behavioral study demonstrating with real words that the advantage of the hand over the mouth is more marked with concrete than with abstract concepts, likely because of the activation of linguistic information with abstract concepts.

## Introduction

We all know what is a “hat,” and what is “justice.” However, we would likely agree that the knowledge of what is “justice” is more complex than knowing what a hat is, also because we can more easily represent the referent of a “hat” through the senses, as vision and touch; furthermore, even if all our concepts have marked inter-individual differences, the way people of our culture represent a hat is likely more homogeneous than the way they represent justice; finally, we likely change over time our notion of “hat” less than our notion of “justice.” Even if we do not think they are dichotomously opposed, we consider “hat” and “justice” as two quite good examples, respectively, of concrete and of abstract concepts.

While the importance of the challenge to understand how we represent concepts has been recognized even by ancient philosophers, the debate on how abstract concepts, such as “justice” and “career,” are represented has recently become particularly hot, as testified by recent reviews and special topics (for recent reviews, see Pecher et al., 2011; Shallice and Cooper, 2013; Borghi and Binkofski, 2014; Dove, 2016; Reilly et al., 2016; for special issues see Borghi and Pecher, 2011, Frontiers in cognition; Tomasino and Rumiati, 2013, Frontiers in Human Neuroscience). This is likely due to the raise and development of embodied and grounded (from now on EG) theories, according to which cognition is grounded in our experiences, sensorimotor system, and bodily states (Barsalou, 2008; Borghi and Caruana, 2015). After their initial development, in the second half of the nineties', in the last years EG theories have been supported by an increasing amount of evidence and have seen an exponential growth in the fields of conceptualization and language comprehension (Gallese and Lakoff, 2005; Gallese, 2008; Chatterjee, 2010; Gentner, 2010; Barsalou, 2012, 2016; Bergen, 2012; see for special issues Borghi and Pecher, 2011; Cappa and Pulvermüller, 2012; Dove, 2015). However, this evidence is mainly focused on representation of objects and actions, while compelling evidence on different kinds of abstract concepts and, most importantly, a unitary EG view explaining all kinds of abstract concepts is still missing, as the brief review below will show. One of the reason underlying this lack is obvious, even if not trivial: demonstrating that abstract concepts as “justice” or “freedom” are grounded in the sensorimotor system is not as straightforward as demonstrating that the concept of “cup” activates the motor system. It is therefore widely recognized that solving the problem of abstract concepts representation would represent a major leap forward for EG cognition perspective.

Still, the benefits of a deeper comprehension of how abstract concepts are represented would not concern only EG theories. The alternative to EG theories are distributional views of meaning. According to these theories abstract concepts can be equated to concrete ones, since the meaning of both kinds of concepts is given by their associations in a semantic network (e.g., Lund and Burgess, 1996; Landauer and Dumais, 1997). However, in our view distributional views are not able to fully solve the problem of conceptual representation, since they are affected by the symbol grounding problem (Harnad, 1990): how can we represent a concept without ever having an idea of its referent?

To address the challenge to explain abstract concepts in the last 10–15 years many proposals on abstract concepts have been presented, which start from an embodied background. The two traditional and yet influential theories on abstract concepts, proposed back in the eighties respectively by Paivio (1986) and Schwanenflugel et al. (1988, 1992), ascribe, respectively, the disadvantage in processing and recall of abstract over concrete words either to their lower degree of imageability, hence to the impossibility to retrieve both verbal and imagistic information for abstract concepts, or to the reduced availability of contexts for abstract concepts. While recent proposals on abstract concepts have taken inspiration from Paivio's dual coding theory (DCT) and have proposed it in a novel version, the context availability theory (CAT) has had less fortune, also due to some recent empirical disconfirmation: for example, Connell and Lynott (2012) have shown that concepts characterized by higher perceptual strength, which CAT would categorize as concrete, evoke a higher number of contexts compared to concepts with low perceptual strength. Moffat et al. (2015) confirmed the importance of contextual availability for concepts, but questioned that the relevance of this construct is unique to abstract concepts.

Recent EG theories on abstract concepts can be divided into two kinds: those that do not focus on the difference between concrete and abstract concepts, trying to demonstrate that both concrete and abstract concepts are grounded in perception and action, and those that consider abstract and concrete concepts different, even if not dichotomously opposed.

Examples of evidence favoring the view proposing that abstract and concrete concepts do not differ is obtained thanks to the Action-sentence Compatibility Effect (ACE) and the approach-avoidance effect. The ACE effect shows that participants are faster when the forward vs. away movement they are required to perform is congruent with the movement implied by both abstract and concrete sentence (“give/take the pizza” vs. “give/take the news”; Glenberg et al., 2008a,b; Guan et al., 2013). In a similar vein, evidence on approach-avoidance has shown that participants tend to attract positive objects and entities and to reject negative ones with both concrete and abstract concepts (Chen and Bargh, 1999; see for a recent overview Phaf et al., 2014 and for a study on oral approach-avoidance Topolinski et al., 2014). Even if this evidence is interesting, it is clearly confined to specific cases (e.g., use of transfer sentences, use of words with a clear positive vs. negative valence).

More crucial for the present paper are proposals that, even if they do not assume that abstract and concrete concepts represent a dichotomy, try to explain the numerous differences between concrete and abstract concepts. The older and most influential one is the Conceptual Metaphor Theory (Lakoff and Johnson, 1980), according to which we understand the meaning of abstract words mapping them to concrete words: for example, we understand the meaning of “category” referring to the concrete concept of “container” (Boot and Pecher, 2011), or the abstract concept of “time” relying to the more concrete notion of “space” (e.g., Santiago et al., 2007; Casasanto and Boroditsky, 2008; Lai and Boroditsky, 2013). A lot of both linguistic and behavioral compelling evidence has been provided in the last years in support of the CMT (see an overview by Winter et al., 2015). This theory has the advantage to focus on a mechanism rather than on the content of specific abstract concepts. However, it remains to be understood to what extent it is generalizable to those abstract concepts the concrete counterpart of which is not obvious, as “fantasy.” Furthermore, its developmental course is not obvious, since metaphors are learned by children later than abstract concepts, and children's comprehension of metaphors remains quite poor until 8–10 years of age (Dove, 2009). According to the recently proposed Affective Embodiment Account (AEA; Kousta et al., 2011; Vigliocco et al., 2013, 2014), abstract concepts would be characterized by the fact that they evoke mostly emotional experience compared to concrete concepts. The theory has recently been supported by behavioral and neuroimaging evidence, and different recent work has highlighted the importance of emotions for abstract concepts (see e.g., Newcombe et al., 2012; Siakaluk et al., 2014). However, such evidence has not always been replicated (e.g., Skipper and Olson, 2014): for example, measuring facial muscle activity a valence effect in the m. corrugator supercilii was found with concrete but not with abstract words (Küenecke et al., 2015). One potential problem of this view is that there is evidence suggesting that emotions represent a subset of concepts which are neither abstract nor concrete, with their own peculiarities (Altarriba et al., 1999; Altarriba and Bauer, 2004; Setti and Caramelli, 2005; Dreyer et al., 2015). A further interesting theory that in our view is full of potentialities was proposed by Barsalou and Wiemer-Hastings (2005) and states that, compared to concrete concepts, abstract ones evoke more social aspects of situations and more introspective properties (see also Zwaan, 2015). The evidence favoring this view is however limited and mostly confined to feature production tasks (see also Borghi et al., 2016); furthermore, the mechanisms underlying abstract concepts representation have not been fully specified. Finally, its generalizability could be limited: for example, introspective elements are likely to occur primarily with mental states and emotional concepts, less with institutional abstract concepts (Roversi et al., 2013). More interesting would be the radical proposal that, the more abstract the concepts are, the more they require introspective mechanisms in action. Further research is thus needed to better clarify these aspects.

The big novelty in the field is represented by multiple representation views (e.g., Barsalou et al., 2008; Dove, 2009; Crutch et al., 2013; Borghi and Binkofski, 2014), and particularly by those that emphasize the importance of both sensorimotor experience and linguistic experience for conceptual representation. The LASS theory, proposed by Larry Barsalou and collaborators (Barsalou et al., 2008; Simmons et al., 2008), has been the first to highlight the importance of language for conceptual representation, even if language is considered more as a shortcut to access the conceptual content than as having an importance *per se*. Dove (2009, 2011, 2016) partly relying on Paivio's view has emphasized the importance of language for abstract concepts representation. His theoretical proposal is very close to the WAT proposal we are focusing on here; the main departure is that, according to Dove, language provides an amodal medium of thought, while we do not see the necessity that such a system is amodal and not grounded in sensorimotor systems.

Here we will focus on the WAT (Words As social Tools) proposal (Borghi and Cimatti, 2009; Borghi and Binkofski, 2014), which can be considered as an embodied and grounded multiple representation view. According to this view, both concrete and abstract concepts are grounded in the sensorimotor system. However, abstract concepts would activate more the linguistic and social neural system, because the linguistic and social experiences are particularly relevant for their acquisition. In support of this idea, the literature on Modality of Acquisition (Wauters et al., 2003) shows that more concrete words are typically acquired perceptually, while with an increase in words complexity and abstractness and in children's age the linguistic acquisition modality becomes progressively more relevant. Due to the importance of linguistic experience for abstract concepts, according to WAT abstract concepts would be more affected by cross-linguistic differences compared to concrete ones. As to brain representation, linguistic networks related both to language comprehension and production as well as networks related to social behavior should be more activated by abstract than by concrete concepts. This activation of language should lead to a higher involvement of the mouth during conceptual processing, in line with the view that both language comprehension and production involve the motor system and are two faces of the same coin (D'Ausilio et al., 2009, 2012; Lieberman, 2009).

The experiment we will report focuses precisely on the mouth activation during abstract concepts comprehension. If language plays a major role for the representation of abstract concepts, then we predict that this activation of linguistic experience has an embodied counterpart, and that it leads to a higher activation of the mouth compared to the hand in the case of abstract concepts.

**Figure 1:**
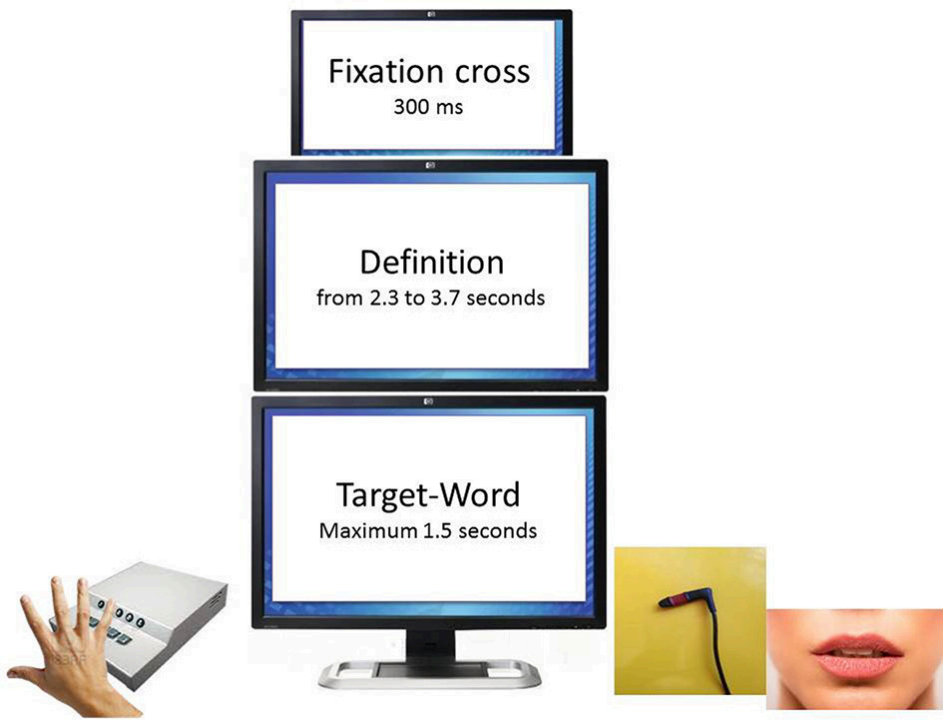
**Experimental paradigm and device**. Participants were required to decide whether the definition was appropriate for the target-word. In order to respond “yes” they had either to press with their dominant hand a key on the response box **(right)** or to press with their teeth the key they held in their mouth **(left)**. The two devices were used by each participant in two different experimental blocks, the order of which was counterbalanced across subjects.

To test this hypothesis we decided to use a task that implies deep conceptual processing. Participants were presented with definitions followed by target-words, and were required to decide whether the definition was correct for the target-word or not. Target-words were concrete and abstract, and definitions also could be more concrete or more abstract. If the definition was correct for the target-word, participants had to press a button; if it was not, they had to refrain from responding (go-nogo paradigm). Critically, in order to record responses we had two different devices: a normal response box, with a key at the center that participants had to press with the dominant hand, and a key typically used for paraplegic patients, that participants kept among their teeth and pressed with a gesture similar to that of biting (see Figure 1). We could not use the same key in both cases since the key for paraplegic patients would have been very hard to press with the fingers.

We predicted to replicate the concreteness effect: in keeping with most of the literature (but see Kousta et al., 2011, for an opposite abstractness effect, and Barca et al., 2002, who did not find the effect), we predicted that abstract words would be processed slower than concrete ones.

More crucially, we predicted that, if it is true that abstract concepts activate linguistic information, hence the mouth, in order to process their meaning, then we should find an interaction between the kind of word and the effector used to respond. Specifically, we expected a facilitation with the mouth responses with abstract concepts compared to concrete concepts.

## Materials and methods

### Participants

Twenty-nine students of the University of Bologna (not involved in pretests; 17 females and 12 males; mean age: 22.9; range: 18–33 years) participated in the experiment. All participants were Italian native speakers, they were right-handed, had normal or corrected-to-normal vision, normal hearing, and no history of neurological/psychiatric disorders or brain damage based on self-report. All participants gave informed consent to participate in accordance with the guidelines of the Ethical Committee of the University of Bologna and volunteered for their participation. The study was approved by the Ethical Committee of the University of Bologna.

### Materials

Materials were composed by two groups of stimuli: definitions, used as primes, and words, used as targets.

We first selected 15 concrete and 15 abstract words from the database of Italian words by Della Rosa et al. (2010), for a total of 30 target-words. In order to distinguish concrete and abstract stimuli we considered various dimensions: concrete and abstract words significantly differed not only according to the Abstractness and Concreteness dimensions, but also according to Imageability, Acquisition Modality, Age of Acquisition and Contextual Availability (*p*s < 0.01). The Average Length of the words and the words Familiarity did not differ across concrete and abstract categories (*p*s < 0.1). Overall, concrete words were more concrete, less abstract, more imageable, more often perceptually rather than linguistically acquired, earlier acquired, and they activated more contexts compared to abstract ones. We decided to use these dimensions to polarize the two categories. Furthermore, since in the literature it is in some cases assumed, on the basis of ratings, that emotions represent a subset of abstract words (e.g., Kousta et al., 2011), while some evidence shows that emotions are processed differently from both concrete and abstract words (Altarriba et al., 1999; Altarriba and Bauer, 2004), we decided to avoid including strongly emotionally valenced words, as “fault” (“colpa”), “relief” (“sollievo”), “disease” (“disagio”), from the sample of abstract words.

For each of the selected words we created two different definitions, a more abstract and a more concrete one, for a total of 60 definitions. We distinguished the two kinds of definitions using the following criteria. Concrete definitions typically described the content of the word using perceptual relations, for example referring to their color and shape (e.g., “flag”: “the Italian one is red, white, and green”), and/or thematic relations, for example referring to specific situations and events (e.g., “career”: “When you get a promotion at work”). Abstract definitions were instead more general and scientific, and were typically expressed through taxonomic relations: e.g., “cock” “Pet bird, it belongs to the family of chickens.”

Definitions were pretested in order to ensure that they differed in degree of abstractness. All the definitions were submitted to a rating task performed by an independent group of 20 students of the University of Bologna. Participants were required to decide, using a seven point scale, whether the definition they read could be considered as more abstract or more concrete. A T Student for paired samples on the average ratings of all definitions (*N* = 20) was only marginally significant, *t*_(19)_ = 1.746, *SE* = 13.515, *p* = 0.097. In the cases in which the distinction between the two definitions was not sufficiently marked, i.e., the two average ratings did not differ, the two definitions were thus modified by the experimenters in order to render them more clearly different. Specifically, we reformulated the concrete definition of “statue” and the abstract definitions of “justice,” “logics,” and “duty.” A further T Student for paired samples on the average ratings of all definitions except the reformulated ones (*N* = 16) showed that the selected definitions significantly differed in abstractness, *t*_(15)_ = 2.800, *SE* = 13.481, *p* = 0.013.

Among the original 30 words we selected 20 test words; each word was accompanied by two definitions. The remaining 10 words and 20 definitions were used as fillers (see Table 1). Overall we had a total of 129 stimuli, each composed by a definition and a word. There were 40 critical stimuli, each of which presented a correct combination of a definition and a word; each of them was repeated twice, once for each of the two experimental blocks. Nine stimuli were used for the training and 40 stimuli, which presented wrong combinations of definitions and words, were used as “fillers.” To ascertain that the selected words were not highly emotional ones, we asked 16 participants to decide using a 7-points Likert scale to what extent each word referred to the emotional sphere. Participants were presented with the 20 selected words and with further 16 words from the same database (Della Rosa et al., 2010) that we considered emotional (e.g., “love,” “anxiety”). The 20 selected words significantly differed from the emotional ones, *t*_(34)_ = 7.157, *SE* = 42.727, *p* < 0.001. The by-items ANOVA on the three kinds of words (selected abstract words, selected concrete words, and emotional words) was also significant, *F*_(2, 33)_ = 100.997, *R*^2^ = 0.860, *p* < 0.001). A follow-up analysis of the simple effects, with the Bonferroni adjustment, revealed that both concrete and abstract words scored lower in emotional value than emotional words (*p*s < 0.001), thus confirming our choice. The abstract and concrete selected words differed according to Concreteness, Abstractness, Imageability, Acquisition Modality, Age of Acquisition, and Contextual Availability (respectively, Concreteness, *t*_(18)_ = 9.513, *SE* = 15.375, *p* < 0.001; Abstractness: *t*_(18)_ = 24.239, *SE* = 16.870; Imageability, *t*_(18)_ = −26.090, *SE* = 16.39715, *p* < 0.001; Modality of Acquisition: *t*_(18)_ = 12.360, *SE* = 22.968; Age of Acquisition: *t*_(18)_ = −7.315, SE = 24.375, *p* < 0.001; Contextual Availability: *t*_(18)_ = −13.042, *SE* = 16.147, *p* < 0.001, *p* < 0.001, *p* < 0.001; while they did not differ in Number of letters and in Familiarity [Familiarity: *t*_(18)_ = −0.443, *SE* = 8.348, *p* = 0.663; Number of Letters: *t*_(18)_ = 0.596, *SE* = 0.48648, *p* = 0.545].

**Table 1:** **The stimuli**.

**SELECTED WORDS AND DEFINITIONS**			
**Word**	**Kind of word**	**Definition**	**Kind of definition**
Gallina hen	Concrete	Il suo verso è “coccodè,” ha due zampe e si trova nelle fattorie. /Its verse is “cackle,” it has two legs and is located in farms.	Concrete
		Uccello domestico, appartiene alla famiglia dei polli. /Pet bird, it belongs to the family of chickens.	Abstract
Sabbia sand	Concrete	Può formare fondali marini e spiagge bianche o dorate. /It can form sea backdrops and white and golden beaches.	Concrete
		Roccia costituita da granelli di altre rocce./Rock composed of grains of other rocks.	Abstract
Ghiaccio ice	Concrete	Forma gli iceberg o si trova a cubetti nei cocktail. /It shapes icebergs or it can be found in cubes in cocktails.	Concrete
		Elemento formato dall'acqua a zero gradi e allo stato solido. /Element formed by water at zero degrees and in the solid state.	Abstract
Cappello hat	Concrete	Può essere di paglia o a cilindro e viene portato in testa. /It can be of straw, it can be a cylinder and it is typically worn on the head.	Concrete
		Accessorio d'abbigliamento che si indossa in testa. /Accessory clothing worn on the head.	Abstract
Stivale boot	Concrete	Calzatura alta ed elegante, anche adatta al maltempo. /Tall and elegant footwear, also suitable for bad weather.	Concrete
		Vi rassomiglia molto la forma dell'Italia. /It is very similar to the shape of Italy.	Abstract
Fontana fountain	Concrete	La trovi nelle piazze, puoi bere l'acqua che ne sgorga. /You can find it in the streets/squares, you can drink the water that gushes.	Concrete
		Costruzione ornamentale da cui fuoriescono getti d'acqua. /Ornamental building from which produce water jets.	Abstract
Statua statue	Concrete	Raffigura oggetti, animali, eroi, è in pietra, bronzo o altri materiali. /It depicts objects, animals, heroes, it is made of stone, bronze or other materials.	Concrete
		Opera di scultura a tutto tondo, in marmo o bronzo. /Sculpture work in the round, in marble or bronze.	Abstract
Pennello brush	Concrete	Lo tiene tra le dita il pittore per dipingere. /The painter holds it among his/her fingers in order to paint.	Concrete
		Strumento per stendere vernice o per dipingere. /Instrument used to spread paint or to paint.	Abstract
Pistola pistol	Concrete	Arma da fuoco dotata di grilletto, che si impugna con una mano sola. /Firearm with trigger, that you hold with one hand.	Concrete
		Arma da fuoco a canna corta dotata di caricatore e grilletto. /Short-barreled firearm with a magazine and trigger.	Abstract
Bandiera flag	Concrete	Quella italiana è di colore rosso, bianco e verde. /The Italian one is red, white and green colored.	Concrete
		Può simboleggiare una nazione o una squadra. / It can symbolize a nation or a team.	Abstract
Inizio beginning	Abstract	Il momento in cui si apre una partita, un gioco o una storia. /The moment in which a game or a story starts.	Concrete
		Il punto di partenza, il primissimo momento./The starting point, the very first moment.	Abstract
Ragione reason	Abstract	La segui quando prendi una decisione senza basarti sui sentimenti. /You follow it when you take a decision without relying on your feelings.	Concrete
		Intelletto, facoltà che distingue gli uomini dalle bestie. /Intellect, quality distinguishing humans from beasts.	Abstract
Critica criticism	Abstract	Si fa quando si scrive un saggio per giudicare l'opera di un artista. /You make it when you write an essay to evaluate the work of an artist.	Concrete
		Facoltà di giudicare obiettivamente opere o persone. /Ability to objectively judge works or people.	Abstract
Motivo motive	Abstract	Chi è assente ne ha sempre uno buono per giustificarsi. /Who is absent has always a good one to justify himself/herself.	Concrete
		Causa, ragione che spinge a compiere una determinata azione. /Cause, reason that induces somebody to perform a given action.	Abstract
Coscienza consciousness/conscience	Abstract	Se la sente sporca chi commette una cattiva azione. /The person who commits a bad action feels it dirty.	Concrete
		Consapevolezza che i soggetti hanno di sé stessi e del mondo. /Awareness that subjects have of themselves and of the world.	Abstract
Dovere duty	Abstract	Lo compie un soldato che esegue i suoi ordini. /A soldier executing the orders accomplishes it.	Concrete
		Legge morale che impone l'esecuzione degli obblighi morali o legali. /Moral law imposing the execution of moral or legal obligations.	Abstract
Giustizia justice	Abstract	Punire i colpevoli, risarcire i danneggiati, dare a ognuno ciò che gli spetta. /To punish the guilty, to repair the damage, to give everyone his/her due.	Concrete
		La virtù data dalla volontà di rispettare il diritto di tutti. /The virtue given by the willing to respect the rights of everyone.	Abstract
Carriera career	Abstract	Si fa quando al lavoro ottieni una promozione. /You make it when you obtain a promotion at work.	Concrete
		Il progresso nel proprio campo lavorativo. /The progress in one's own working field.	Abstract
Fiducia trust	Abstract	Si ha quando ti lasci cadere indietro sapendo che qualcuno ti prenderà. /You have it when you let yourself fall back knowing that somebody will take you.	Concrete
		Senso di sicurezza che deriva dalla stima riposta in qualcuno. /Security feeling deriving from the estimate placed in someone.	Abstract
Logica logics	Abstract	La usi quando da un indizio fai un ragionamento corretto. /You use it when from a clue you make a correct reasoning.	Concrete
		Disciplina filosofica che studia le leggi del ragionamento. /Philosophical discipline that investigates the laws of reasoning.	Abstract

### Procedure

We used a go-nogo paradigm: Participants were instructed to read the definition followed by the word and to press a button if the definition was appropriate for the word. If the definition was not appropriate, they had to refrain from responding.

The experiment included two experimental blocks, the presentation order of which was counterbalanced across participants. The trials in the two blocks were the same, but in one block responses had to be given with the hand, in the other with the mouth. Each block was preceded by some training trials, in order to allow participants to practice with the new button, especially the button to press with the teeth: three trials preceded the first block, and six training trials preceded the second block. Each experimental block was composed by 60 trials, of which 40 were correct combinations, and 20 were incorrect combinations, i.e., fillers; all trials were presented in random order. Participants were randomly assigned to one of the two groups.

Participants were individually tested in a quiet laboratory room (Emcolab, University of Bologna). Only the participant and the experimenter were present in the room; after the training the experimenter sat outside the participant's sight in order to avoid any interference with the task.

Testing took place on a PC running EPrime2 Professional software. Participants sat on a comfortable chair in front of a computer screen, at a distance of about 60 cm. They read the written instruction describing the experiment on the computer screen. In no case further instruction from the experimenter were needed; he only needed to clarify to them how to use the button for the mouth responses.

Each trial began with a centered black fixation cross for 300 ms, followed by presentation of a definition. Definitions remained on the screen for a time varying from 2.3 to 3.7 s, depending on the number of syllables each definition included. Once the definition disappeared, the word appeared; it remained on the screen until the response, or for a maximum of 1.5 s. After 1 s the next trial started. Participants were asked to decide if the definition and the word matched and to indicate their decision by pressing a key. They were invited to respond as quickly and accurately as possible as their response time and accuracy were being measured. Depending on the block, the key had to be pressed either with the hand, or with the mouth.

## Results

Responses to filler sentences and incorrect responses (6.51%) were discarded. Data were trimmed as follows: RTs faster/slower than the overall participant mean minus/plus 2 SD (4.007%) were excluded from the analyses.

Kendall's tau-b and Spearman correlation coefficients were computed between the mean Response Times (RTs) of the correct responses and the values of the dimensions obtained from the database of Della Rosa et al. (2010) on the basis of which our target words were selected. Results showed a positive correlation between RTs and Abstractness, *r* = 0.540, *p* = 0.001; *r* = 0.770, *p* < 0.001; RTs and Age of Acquisition: *r* = 0.417, *p* = 0.010; *r* = 0.649, *p* = 0.002, and RTs and Modality of Acquisition: *r* = 0.474, *p* = 0.004; *r* = 0.699, *p* = 0.001, indicating that RTs were longer, the more abstract words were, the later they had been acquired and when they were acquired perceptually rather than through linguistic modalities. RTs showed instead a significant negative correlation with Concreteness, *r* = −0.451, *p* = 0.006; *r* = −0.701, *p* = 0.001, Contextual Availability, *r* = −0.512, *p* = 0.002, *r* = −0.724, *p* < 0.001, and Imageability, *r* = −0.474, *p* = 0.004; *r* = −0.690, *p* = 0.001. Basically RTs were faster the more words were concrete, evoked a higher number of contexts, and were more imageable. The correlations between RTs and words Familiarity and number of Letters were not significant, respectively: *r* = −0.026, *p* = 0.871, *r* = −0.041, *p* = 0.863; *r* = −0.138, *p* = 0.436, *r* = −0.188, *p* = 0.427.

Mean Response Times of the correct responses were then submitted to a Repeated Measures Analysis of Variance (ANOVA) with Definition (Abstract vs. Concrete), Word (Abstract vs. Concrete), and Effector (Hand vs. Mouth) as the within-subject factors (F_1_). A further within-items ANOVA (F_2_) was performed, with Definition (Abstract vs. Concrete), and Effector (Hand vs. Mouth) as the within-item factors and Word (Abstract vs. Concrete) as the between-item factor.

**Figure 2:**
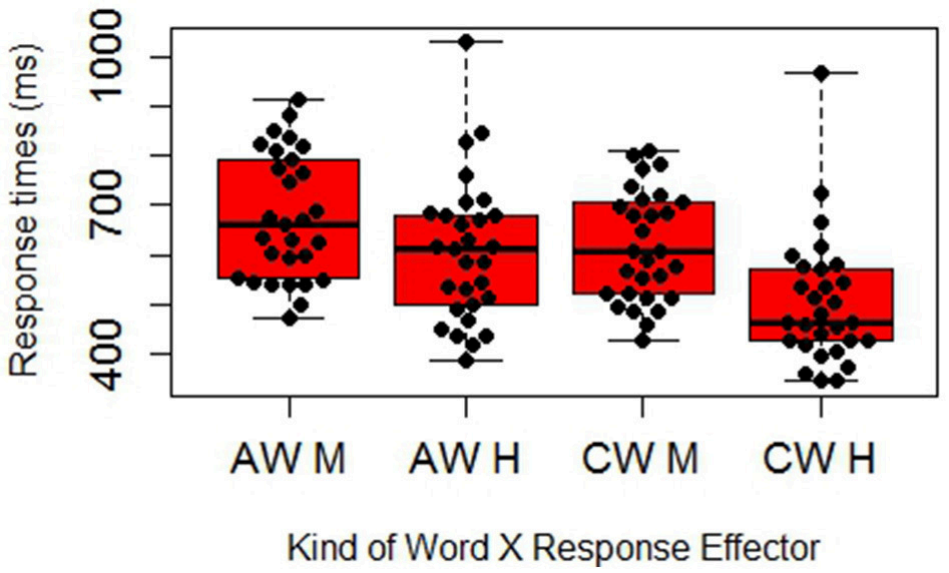
**Boxplot and beeswarm graph of response times, showing the interaction between the kind of word (AW, Abstract Words; CW, Concrete Words) and the effector (M, Mouth; H, Hand)**.

**Figure 3:**
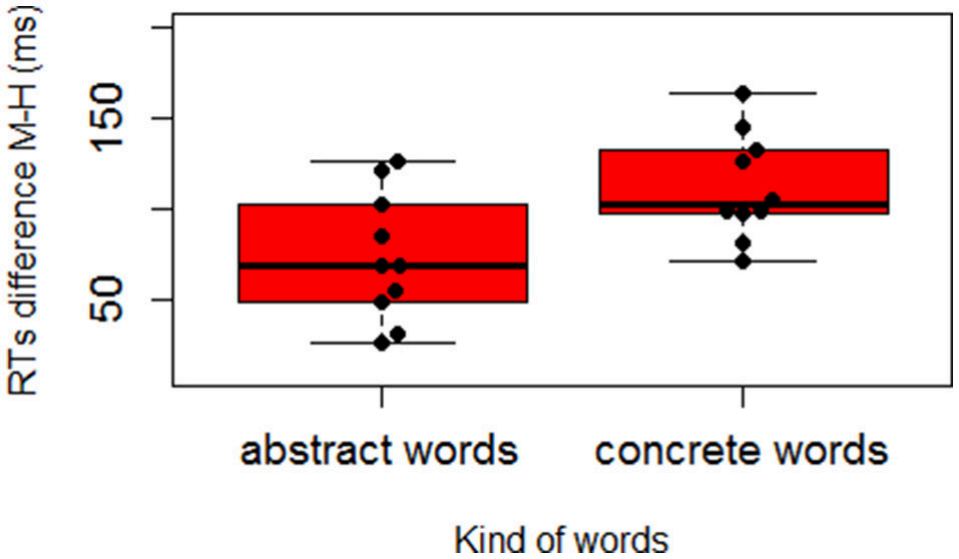
**Boxplot and beeswarm graph plotting the difference in response times between the Mouth and the Hand response (Δ_M–H_ = M_M_ − M_H_) for Concrete and Abstract words**.

**Figure 4:**
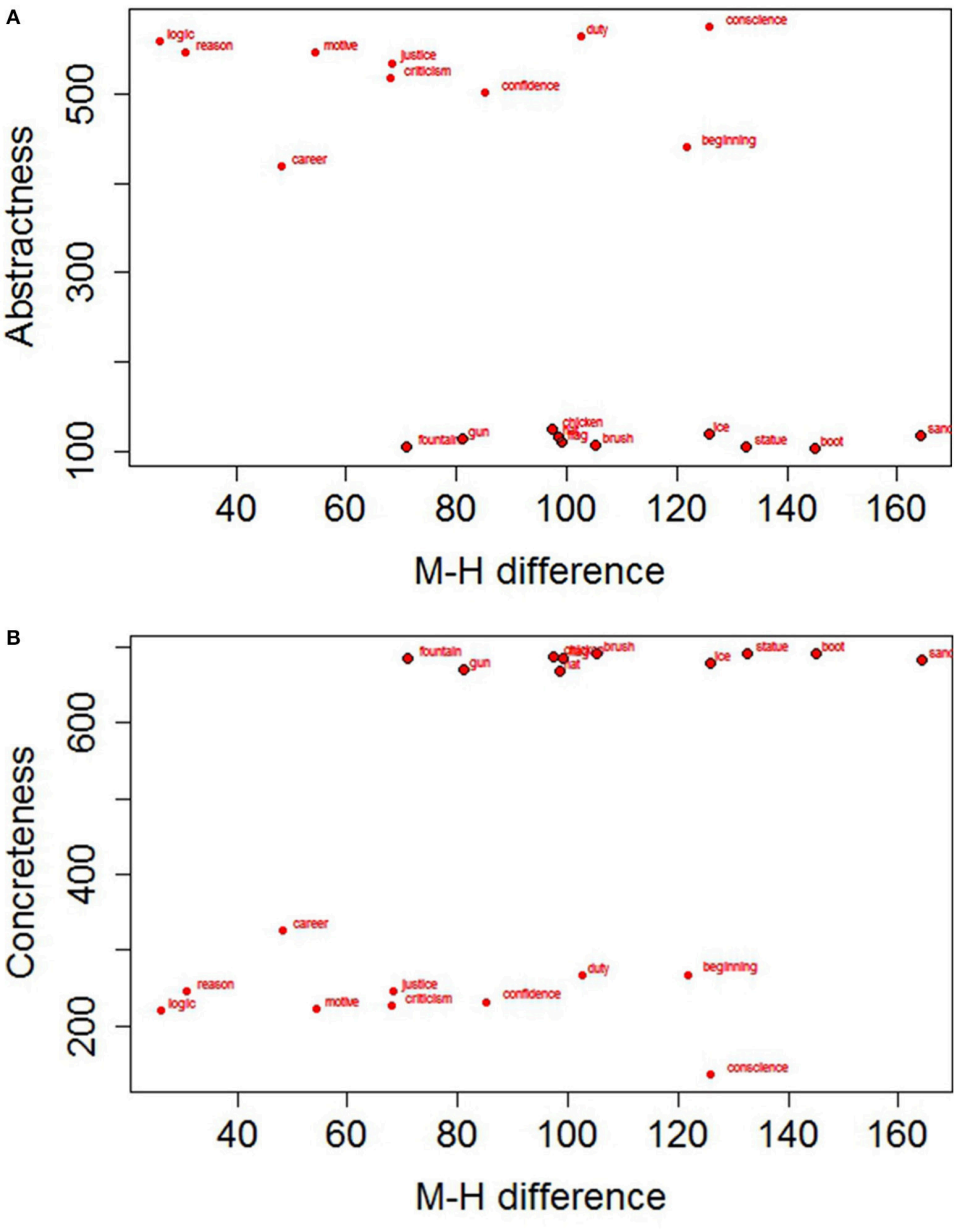
**Hand-Mouth difference scores in response times plotted as a function of Abstractness (A) and Concreteness (B) values**. The points with a black border refer to Concrete Words.

All main effects were significant: as predicted, Abstract definitions (*M* = 623) were processed slower than Concrete ones (*M* = 578), $F_{\left(1,28\right)}^{1}$ = 37.344, *R*^2^ = 0.571, *p* < 0.001; *F*^2^_(1, 18)_ = 5.889, *R*^2^ = 0.247, *p* = 0.026, and Abstract target-words (*M* = 641) were processed slower than concrete ones (*M* = 560), $F_{\left(1,28\right)}^{1}$ = 99.806, *R*^2^ = 0.781, *p* < 0.001; $F_{\left(1,\;18\right)}^{2}$ = 20.116, *R*^2^ = 0.528, *p* < 0.001. In order to verify whether the advantage of abstract over concrete words was due to differences in frequency we controlled word frequency using CoLFIS, a lexical database of written Italian (Bertinetto et al., 2005). We performed two T Student's analyses for independent samples considering the overall frequency of our target-words both as word forms and as lemmas. Both analyses revealed that the selected abstract words were overall more frequent than the selected concrete ones, *t*_(18)_ = 3.779, *SE* = 66.156, *p* < 0.01; *t*_(18)_ = 4.018, *SE* = 68.015, *p* < 0.01. This result clearly rules out that the concreteness effect we found was due to differences in word frequency rather than in word meaning: if it was due to frequency, the pattern should have been reversed, with a facilitation for abstract over concrete words. Finally, responses with the mouth (*M* = 647) were slower than responses with the hand (*M* = 555), $F_{\left(1,28\right)}^{1}$ = 11.833, *R*^2^ = 0.297, *p* = 0.002; $F_{\left(1,18\right)}^{2}$ = 163.526, *R*^2^ = 0.901, *p* < 0.001. The interaction between Definition and Word was also significant (see Figure 2), $F_{\left(1,28\right)}^{1}$ = 4.274, *R*^2^ = 0.132, *p* = 0.048; $F_{\left(1,18\right)}^{2}$ = 1.003, *R*^2^ = 0.053, *p* = 0.330: the advantage of concrete definitions over abstract ones was more pronounced with abstract words than with concrete words. This result is consistent with the higher difficulty of abstract words and the need to ≪ground≫ them in concrete experiences and situations. More crucially, the interaction between the effector and the target-word was significant, $F_{\left(1,\;28\right)}^{1}$ = 6.651, *R*^2^ = 0.192, *p* = 0.015; $F_{\left(1,\;18\right)}^{2}$ = 7.194, *R*^2^ = 0.286, *p* = 0.015. The interaction was due to the fact that, whereas the hand responses had an overall advantage, with abstract words the advantage of the hand over the mouth responses was less pronounced. To better understand the pattern of our data, we performed T Student's analyses for paired samples (in the analyses by participants, indicated by *t*^1^) and for independent and paired samples (in the analyses by concepts, indicated by *t*^2^; see Tables 2A,B). We found that, while the advantage of concrete over abstract words was significant both with the hand and with the mouth device, the difference between mouth and hand device was more marked when considering concrete concepts than when considering abstract ones. The analyses and the effect sizes (Cohen's d) revealed indeed that the difference between abstract and concrete words was highly significant for hand responses, $t_{\left(28\right)}^{1}$ = 9.499, *SE* = 10.909, *p* < 0.001, *D* = 0.727; $t_{\left(18\right)}^{2}$ = 5.180, *SE* = 19.776, *p* < 0.001, *D* = 2.317, and significant but slightly less pronounced for mouth responses: $t_{\left(28\right)}^{1}$ = 4.697, *SE* = 12.671, *p* < 0.001, *D* = 0.484; $t_{\left(18\right)}^{2}=$ 3.184, *SE* = 19.979, *p* < 0.01, *D* = 1.424. Crucially for our hypotheses, further T student analyses showed also that the difference between hand and mouth responses was more marked within concrete concepts than with abstract concepts (*p* < 0.001 vs. *p* = 0.023): abstract concepts: $t_{\left(28\right)}^{1}=$ 2.412, *SE* = 29.046, *p* = 0.023, *D* = 0.510; $t_{\left(9\right)}^{2}=$ 6.571, *SE* = *, p* < 0.001, *D* = 1.343, concrete concepts: $t_{\left(28\right)}^{1}$ = 4.206, *SE* = 27.143, *p* < 0.001, *D* = 0.932; $t_{\left(9\right)}^{2}$ = 12.099, *SE* = 9.255, *p* < 0.001, *D* = 2.321. Finally, to test directly whether the differences between effectors of abstract and concrete targets differed significantly from another, we computed the difference score Δ_M-*H*_ between mouth and hand response times for each participant and for each target-word:

$$\begin{array}{l} \Delta_{\text{M-H}}={\text{M}}_{\text{M}}–{\text{M}}_{\text{H}} \end{array}$$

where M_M_ is the average response time with the mouth for each participant/word, and M_H_ is the average response time with the hand for the same participant/word. We then performed two analyses on the difference scores: a T Student for paired samples on participants and a T Student for independent samples on items. Both analyses were significant, $t_{\left(28\right)}^{1}$ = −2.579, *SE* = 17.103, *p* = 0.015, *D* = 0.295; $t_{\left(18\right)}^{2}$ = −2.682, *SE* = 14.477, *p* = 0.015, *D* = −1.199. The results support our prediction of a higher activation of the mouth with abstract concepts leading to a less pronounced difference between mouth and hand responses with abstract concepts (*M* = 70.06, *SD* = 29.05) than with concrete ones (*M* = 114.17, *SD* = 27.14; see Figure 3). Figures 4A,B further show how the Hand-Mouth difference scores vary as a function of the degree of abstractness and concreteness of each selected word. Figures 3, 4A,B clearly show that the difference we found is not simply due to a higher activation of the hand with abstract words but both to a higher activation of the mouth with abstract words and of the hand with concrete words.

**Table 2A:** **Interaction effector × word**.

**MEANS AND STANDARD DEVIATIONS**		
**Hand responses:abstract vs. concrete words**		
	**Abstract words**	**Concrete words**
Participants	Mean = 606.476	Mean = 502.856
	*SD* = 145.702	*SD* = 131.377
Concepts	Mean = 604.159	Mean = 501.710
	*SD* = 57.418	*SD* = 24.784
**Mouth responses:abstract vs. concrete words**		
	**Abstract words**	**Concrete words**
Participants	Mean = 676.538	Mean = 617.025
	*SD* = 127.923	*SD* = 112.253
Concepts	Mean = 677.307	Mean = 613.788
	*SD* = 43.575	*SD* = 45.748
**Abstract words: hand vs. mouth responses**		
	**Mouth**	**Hand**
Participants	Mean = 676.538	Mean = 606.476
	*SD* = 127.9238	*SD* = 145.702
Concepts	Mean = 677.307	Mean = 604.159
	*SD* = 43.575	*SD* = 57.418
**Concrete words: hand vs. mouth responses**		
	**Mouth**	**Hand**
Participants	Mean = 617.025	Mean = 502.856
	*SD* = 112.253	*SD* = 131.377
Concepts	Mean = 613.788	Mean = 501.710
	*SD* = 45.748	*SD* = 24.784
**Hand-Mouth difference scores**		
	**Abstract words**	**Concrete words**
Participants	Mean = 70.062	Mean = 114.169
	*SD* = 156.417	*SD* = 146.167
Concepts	Mean = 73.148	Mean = 111.978
	*SD* = 35.203	*SD* = 29.266

**Table 2B:** **Interaction effector × word**.

**T's**		
**Hand responses:abstract vs. concrete words**		
(paired samples)	Participants	*t*_(28)_ = 9.499, *SE* = 10.909, *p* < 0.001, D = 0.727
(independent samples)	Concepts	*t*_(18)_ = 5.180, *SE* = 19.776, *p* < 0.001, D = 2.317
**Mouth responses: abstract vs. concrete words**		
(paired samples)	Participants	*t*_(28)_ = 4.697, *SE* = 12.671, *p* < 0.001, D = 0.484
(independent samples)	Concepts	*t*_(18)_ = 3.184, *SE* = 19.979, *p* = 0.005, D = 1.424
**Abstract words: hand vs. mouth responses**		
(paired samples)	Participants	*t*_(28)_ = 2.412, *SE* = 29.046, *p* = 0.023, D = 0.510
(paired samples)	Concepts	*t*_(9)_ = 6.571, *SE* = 11.133, *p* < 0.001, D = 1.343
**Concrete words: hand vs. mouth responses**		
(paired samples)	Participants	*t*_(28)_ = 4.206, *SE* = 27.143, *p* < 0.001, D = 0.932
(paired samples)	Concepts	*t*_(9)_ = 12.099, *SE* = 9.255, *p* < 0.001, D = 2.321
**Hand-Mouth difference scores**		
(paired samples)	Participants	*t*_(28)_ = −2.579, *SE* = 17.103, *p* = 0.015, D = 0.295
(independent samples)	Concepts	*t*_(18)_ = −2.682, *SE* = 14.477, *p* = 0.015, D = −1.199

**Figure 5:**
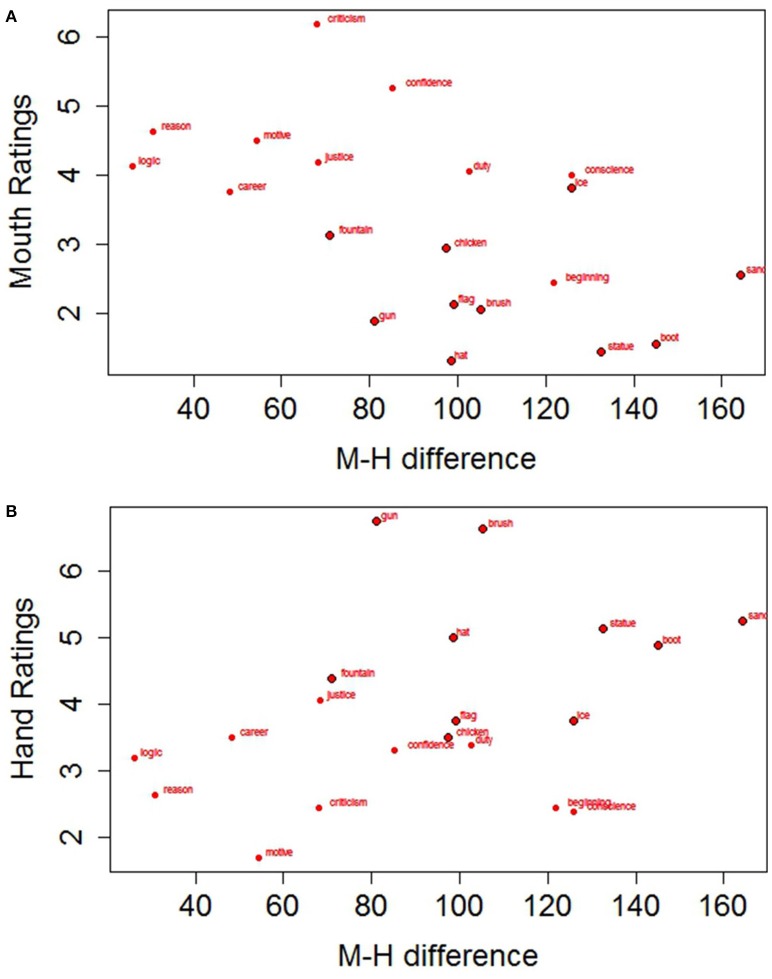
**Hand-Mouth difference scores in response times plotted as a function of Average Mouth Ratings (A) and Average Hand Ratings (B)**. The points with a black border refer to Concrete Words.

In order to further verify to what extent the interaction we found was due more to the higher activation of the hand with concrete concepts or of the mouth with abstract ones, we performed ratings on how the selected words activated the hand and the mouth. We asked 16 independent participants to evaluate using a 7-points Likert scale how much each of the selected words was involved in a possible action with the hand/the mouth. For consistency, we used the same question of Granito et al. (2015) (adapted from Ghio et al., 2013). We presented the same word in two different blocks (Hand-Mouth and Mouth-Hand), the order of which was counterbalanced across participants. We then performed two T Student's for independent samples on both concrete and abstract words, one with the Average Hand Ratings and one with the Average Mouth Ratings. In line with our predictions, average Mouth Ratings were significantly higher with abstract than with concrete words, $t_{\left(18\right)}^{2}$ = 5.055, *SE* = 0.402, *p* < 0.001, *D* = 2.261, while average Hand Ratings were were significantly higher with concrete than with abstract words, $t_{\left(18\right)}^{2}$ = −4.759, *SE* = 42,026, *p* < 0.001, *D* = −2.128 (see Table 2C). In order to understand whether the pattern of RTs results was explained more by the hand or by the mouth average ratings, we performed Kendall's tau-b and Spearman correlations between the mouth and hand ratings and the difference scores in RT. Average Mouth Ratings and Hand-Mouth difference scores in RTs were negatively correlated, *r* = −0.400, *p* = 0.014; *r* = −0.585, *p* = 0.007. The positive correlations between Average Hand Ratings and Hand-Mouth difference scores in RTs instead did not reach significance, suggesting that the RTs pattern was more correlated to the mouth than to the hand ratings, *r* = 0.292, *p* = 0.074; *r* = 0.406, *p* = 0.075 (see Figures 5A,B). The correlation results are thus in keeping with the WAT prediction that mouth activation is stronger with abstract than with concrete words. Two further ANOVAs, one by-participants and one by-items, were performed on the proportion of errors (overall, 6.508%). In the analysis on errors only the main effects of Definition and Word reached significance, and no interaction was significant. Abstract definitions (*M* = 0.905) yielded more errors than Concrete ones (*M* = 0.397), $F_{\left(1,\;28\right)}^{1}$ = 15.782, *R*^2^ = 0.360, *p* < 0.001; $F_{\left(1,\;18\right)}^{2}$ = 5.046, *R*^2^ = 0.219, *p* = 0.037, and Abstract target-words (*M* = 0.940) yielded more errors than concrete ones (*M* = 0.362), $F_{\left(1,\;28\right)}^{1}$ = 3.322, *R*^2^ = 0.402, *p* < 0.001; $F_{\left(1,\;18\right)}^{2}=$ 2.374, *R*^2^ = 0.407, *p* = 0.002. Thus, a concreteness effect was found. Finally, responses with the mouth (*M* = 0.767) yielded slightly more errors than responses with the hand, but the difference did not reach significance in the by-participants analysis (*M* = 0.564), $F_{\left(1,\;28\right)}^{1}=$ 11.833, *R*^2^ = 0.106, *p* = 0.079; $F_{\left(1,\;18\right)}^{2}$ = 10.236, *R*^2^ = 0.363, *p* = 0.005.

**Table 2C:** **Results of ratings on hand and mouth involvement**.

**MEANS AND STANDARD DEVIATIONS**		
**Ratings on hand involvement**		
	**Abstract words**	**Concrete words**
Concepts	Mean = 2.902	Mean = 4.90
	*SD* = 0.701	*SD* = 1.129
**Ratings on mouth involvement**		
	**Abstract words**	**Concrete words**
Concepts	Mean = 4.314	Mean = 2.282
	*SD* = 0.976	*SD* = 0.815
**RESULTS**		
**Ratings on hand involvement**		
(independent samples)	Concepts	*t*_(18)_ = −4.759, *SE* = 0.420, *p* < 0.001, D = −2.128
**Ratings on mouth involvement**		
(independent samples)	Concepts	*t*_(18)_ = 5.055, SE = 0.402, *p* < 0.001, D = 2.261

## Discussion

The results are rather clear, and they support the hypotheses we advanced. In line with the great part of existing literature, we found that concrete words were processed faster than abstract ones (concreteness effect; e.g., Paivio, 1986). We also found a concreteness effect with definitions: more concrete definitions, including more perceptual features and exemplifications, were processed faster than more abstract, taxonomic definitions. The finding of a concreteness effect with definitions not only replicates, but extends results present in current literature. The interaction between definitions and words is consistent with the concreteness effect: since abstract words are more difficult, they benefit more than concrete words of concrete definitions, likely due to the higher need to ≪ground≫ them in concrete situations and experiences. Finally, we also found an overall advantage of the hand over the mouth responses, likely due to the kind of device we had to use to provide mouth responses.

The most crucial and novel result, however, is the interaction we found in response times between the effector used to respond and the kind of words, showing that with abstract words the advantage of the hand over the mouth responses was less pronounced than with concrete ones.

This interaction clearly confirms the hypotheses derived by WAT, according to which linguistic information characterizes mostly abstract concepts, hence leading to the activation of the mouth, while sensorimotor information characterizes primarily concrete ones. WAT directly predicts that linguistic and sensorimotor information are differently distributed across abstract and concrete concepts, and that abstract words activate more the mouth effector; as to concrete words, they should activate sensorimotor information of all kinds. Since concrete words that directly activate the mouth (e.g., food) were not present, the higher activation of the hand with concrete concepts is in line with WAT predictions, given the dominance of the hand in our brain representation. To our knowledge this is the first study in which a difference in the activation of mouth and hand is found with response times using ≪real≫ concrete and abstract words.

This finding is in line with solid fMRI results showing involvement of areas related to language production and comprehension during abstract concepts processing, as the left inferior frontal gyrus and the left middle temporal gyrus (e.g., Wang et al., 2010; Sakreida et al., 2013; Hoffman et al., 2015). Further behavioral studies also confirm the importance of language for abstract concepts. A study on Italian Sign Language (LIS) has shown that abstract concepts exploit linguistic information, taken either from the same sign language, from other sign languages or from spoken languages, to convey their meaning: for example, the abstract concept “vero” (true) is expressed using alliteration, i.e., using a handshape which is also used for the letter V in the manual alphabet (extended index and middle fingers) and adds movement down and to the left of the face (Borghi et al., 2014). Finally, also computational linguistics studies revealed the importance of language for abstract concepts representation. For example, Recchia and Jones (2012) demonstrated that abstract concepts benefit of a semantically rich contexts, while concrete concepts of physically rich contexts.

As to the mouth activation with abstract concepts, previous evidence was obtained teaching adults novel categories and words. Providing adults with an explanation of the characteristics of novel category members led to a higher activation of the mouth with abstract than with concrete novel words (Borghi et al., 2011). Furthermore, the activation of the mouth was higher when participants were taught the name and explained the meaning of new categories (Granito et al., 2015).

To the best of our knowledge the only current evidence demonstrating a preferential activation of the mouth with abstract concepts with “real” abstract words and sentences was obtained through explicit rating tasks. In a study by Ghio et al. (2013) the authors asked participants to rate how much the action described in Mental state, Emotional, and Math-related sentences (≪She memorizes the procedure≫, ≪She feels happy≫, ≪She determines the sum≫) involved the mouth, the hand, and the leg. They found that mental state sentences were highly associated to the mouth, while number-related sentences were related to the hand, likely due to finger-counting experiences, and emotion-related sentences activated both hand and mouth. Granito et al. (2015) asked participants how much the mouth or the hand was involved in a possible action with the target items by using a 7-points Likert scale. Results showed that abstract concepts activated the mouth more than concrete concepts, independently of whether concrete concepts represented an homogeneous and compact category, as in the case of “penguin” (pinguino), or an heterogeneous category composed by profoundly different members, as in the case of “tool” (utensile).

To date, however, no study has revealed that abstract concepts activate the mouth more than concrete concepts with a behavioral task with real words, in which an implicit task was used. To our knowledge the present study is the first behavioral one showing with real words that, even if hands responses are faster with both concrete and abstract concepts, the activation of linguistic information with abstract concepts leads to a smaller difference between mouth and hand responses with abstract than with concrete concepts.

### Why should language be so important for abstract concepts?

Our study demonstrates that, while responses with the hand compared to those with the mouth are facilitated with concrete words, this facilitation is less pronounced with abstract words. This is mostly due to a higher mouth activation with abstract concepts, probably due to the activation of linguistic information.

Once determined that linguistic information is crucial for abstract concepts representation, it is important to understand why. We outline a series of possible explanations, that are not necessarily mutually exclusive.

#### Labels as ≪glue≫ and language as a predictive system

Since abstract concepts refer to more sparse and diverse experiences compared to those of concrete concepts (even of superordinate ones), labels can work as a sort of ≪glue≫ keeping them together (Borghi and Binkofski, 2014). Crucially, words would not only be “pointers” to their referents, but they would contribute in forming the categories. This clearly implies intending language not only as a referential system and a communication system, but a control system that programs human mind manipulating sensorimotor experiences (Lupyan and Bergen, 2016). Recent results have demonstrated that also concrete labels help us in building and forming a category. For example, Lupyan and collaborators (Lupyan, 2012; Boutonnet and Lupyan, 2015) have shown that visual processing is facilitated after hearing the name ≪dog≫ compared to when hearing a sound, such as the sound of a barking dog. Language can provide a means of building predictions: for example, listening to a word can help our visual system to process noisy inputs (Lupyan and Clark, 2015). Evidence obtained mimicking the acquisition of novel categories and words has shown that the contribution of the name to the category formation and the explanation of the word meaning is more crucial for learning abstract words, likely due to the diversity of their referents; and this can explain the strong activation of linguistic information with them. Results with pictures indicate that, when participants are provided with explanations of the word meaning, the mouth is activated (Borghi et al., 2011); furthermore, results with categories the members of which are built through Lego revealed that such activation is more marked when participants have been taught the category name and explained its meaning (Granito et al., 2015).

#### Words as social tools, sign tracking

Consider the difference between “bottle” and “justice”: it is much easier to understand the meaning of “bottle” than of “justice” without the help of others. To understand abstract words we need to rely more on other people's opinions, definitions, and explanations. Consistently, comprehension of the very first abstract words has been related with the development, at 10 and 14 months of age, of crucial social cognition abilities, like that of following the gaze of others and of engaging in joint actions (Bergelson and Swingley, 2013). The importance of the social abilities for the representation of abstract concepts has been well captured by Prinz (2002, 2012) who has spoken of a “word tracking strategy” we would use to access conceptual meaning: abstract words, such as “democracy” would be grasped in part through concrete images, in part through verbal skills. We would namely track definitions used by other authoritative members of our community to help reference (re-enactment). The activation of the mouth in the current experiment, in which participants are given definitions of concepts, is consistent with this account; importantly, we found that concrete definitions are particularly crucial for abstract words, that need more examples and perceptual information to be “grounded.”

#### Language as a way to improve our computational abilities through inner speech

It has been suggested that language has the important role of improving our computational abilities (e.g., Clark, 1998). This improvement can occur through inner speech: the seminal work by Vygotsky has shown that words can become internalized and support our thought processes: for example, speaking to ourselves helps us to better memorize and plan our actions (Vygotsky, 1986). The capability of language to enhance thought processes has been specifically related to abstract concepts by Dove (2009, 2011, 2016). However, according to Dove this potentialities are due to the amodal character of language: he argues that language acquisition would create a new “dis-embodied” semantic system, characterized by many similarities with amodal systems of traditional cognitive science. We think that the computational abilities language offers are particularly crucial for abstract concepts, because they are “hard” words, but we do not see why such a system should be disembodied and amodal. In contrast, we think that the activation of language through inner speech can have an embodied counterpart, the activation of the mouth we found.

### Which are the possible mechanisms underlying the mouth activation?

It remains to be established, which mechanism underlies the activation of the mouth. Two possible mechanisms can cause this activation; importantly, they are not mutually exclusive and also both could be responsible for the mouth activation.

The first mechanism is re-enactment. As argued by WAT and explained in the introduction, abstract concepts are acquired in a peculiar way: since they do not have concrete and clearly bounded referents and because their referents are quite heterogeneous, the labels and explanations of their meaning are crucial to acquire them, hence the linguistic-social experience plays a major role. The re-enactment of this peculiar acquisition experience could be responsible for the mouth activation. The results of the present experiment cannot determine whether this mechanism is in action, since the experiment focuses on existent words that have already been acquired by participants. However, we computed Kendall's tau-b and Spearman correlations between RTs and Modality of Acquisition values in Della Rosa et al.'s (2010) database, and we found that they were highly significant, respectively *r* = 0.474, *p* = 0.004; *r* = 0.699, *p* = 0.001. This suggests that the Modality of Acquisition (perceptual vs. linguistic) contributes in explaining the RTs pattern; whether this is the only cause or only one among the causes of the mouth activation has to be determined through further experiments. This mechanism is compatible both with the idea that labels are a sort of glue and with the sign tracking view: we would namely re-enact both the experiences related to the conceptual referent and the linguistic-social experiences related to the acquisition of the concept. Further evidence on acquisition of novel categories and words supports this view: providing adults with an explanation of the characteristics of novel category members led to a higher activation of the mouth with abstract than with concrete words (Borghi et al., 2011). Furthermore, the activation of the mouth was higher when participants were taught the name and explained the meaning of new categories (Granito et al., 2015). Finally, recent results show that abstract words are more associated to the acoustic modality than concrete ones (Scerrati et al., 2016). Re-enactment would lead to the activation of the mouth since elegant evidence by Fadiga, D'Ausilio and collaborators has shown that both language comprehension and production activate the motor system (e.g., D'Ausilio et al., 2009, 2012).

Another possible mechanism is the re-explanation through inner talk. Due to the fact that abstract words are “hard” words to learn (Gleitman et al., 2005; Gentner, 2006), we might need to re-explain to ourselves their meaning, formulating predictions against which sensory experiences can be assessed, possibly through the mediation of inner talk. This mechanism is compatible with the view of language as a way to improve our computational abilities through inner speech. Supporting evidence comes from the rating study by Ghio et al. (2013) showing that mental state sentences activate preferentially the mouth. The adoption of this re-explanation mechanism, perfectly in line with WAT, is also compatible with the proposal on abstract concepts representation advanced in 2005 by Barsalou and Wiemer-Hastings. It is namely possible that both the longer response times required by abstract concepts and the shorter time required with the mouth than with the hand device are due to the fact that participants re-explain to themselves the meaning of abstract concepts, thanks to introspection, possibly through the use of inner talk. The use of introspection, due also to a specific content (e.g., the high presence of mental words), would then due to an internal mechanism (e.g., talking to oneself?), which could explain the mouth activation.

Since it focuses on words that participants already know, the present experiment does not allow us to determine which of the two mechanism –re-enactment or re-explanation—underlies the activation of the mouth, or if both mechanisms are responsible for it. Further research on development and acquisition is needed, to address this complex question.

## Conclusions

Overall, our results indicate for the first time not only that abstract concepts are grounded in experiences, but also that they are embodied: we found that with abstract concepts the advantage of the hand responses over the mouth responses was less pronounced than with concrete concepts, mainly due to a higher activation of the mouth with abstract concepts. This clearly confirms the prediction of the WAT theory with existent abstract and concrete words.

The result has important implications also for other recent theories that we reviewed in the introduction. It disconfirms embodied views according to which no difference between abstract and concrete concepts exists and it is not predicted by other theories on abstract concepts as the CMT and the AEA view. Furthermore, it suggests how to possibly integrate the multiple representation views and the introspective view.

Consider recent theories that take inspiration from Paivio's view, as the proposal by Dove, and the neural evidence favoring a dual systems account (e.g., Sabsevitz et al., 2005; Binder et al., 2005). According to Dove different levels of embodiment exist, and abstract concepts activate language considered as an amodal medium. The evidence we provide suggests that abstract concepts activate language through an embodied experience, likely a form of inner talk that leads to the preferential activation of a specific effector, the mouth. While it is in line with the view according to which language plays a major role in abstract concepts representation, it also suggests that linguistic information is activated through an embodied mechanism.

Furthermore, the results allow the exploration of the possibility to reconcile WAT and introspective view of abstract concepts (Barsalou and Wiemer-Hastings, 2005). It is namely possible that, due to the complexity of abstract concepts, they evoke introspective information, and that this information is rehearsed and monitored through an embodied mechanism like talking to oneself.

## Author contributions

AB and EZ conceived the experiment. EZ prepared the materials and conducted the experiment. AB analyzed the data and wrote the paper.

### Conflict of interest statement

The authors declare that the research was conducted in the absence of any commercial or financial relationships that could be construed as a potential conflict of interest.
